# Correction: Validation of the Strengths and Difficulties Questionnaire (SDQ) emotional subscale in assessing depression and anxiety across development

**DOI:** 10.1371/journal.pone.0317151

**Published:** 2025-01-02

**Authors:** Jessica May Armitage, Foteini Tseliou, Lucy Riglin, Charlotte Dennison, Olga Eyre, Rhys Bevan Jones, Frances Rice, Ajay K. Thapar, Anita Thapar, Stephan Collishaw

The S2 Fig is uploaded incorrectly. Please view the correct S2 Fig below.

## Supporting information

S2 Fig ROC analyses for emotional subscale predicting Attention Deficit Hyperactivity Disorder or any behavioural diagnosis across development. (TIF)
