# Supplementary material for: Validation of the Strengths and Difficulties Questionnaire (SDQ) emotional subscale in assessing depression and anxiety across development
Source: PLoS One. 2023 Jul 19;18(7):e0288882. doi: 10.1371/journal.pone.0288882 (PMC10355443; doi:10.1371/journal.pone.0288882)

**Fig S1:** ROC analyses for emotional subscale predicting any anxiety diagnosis across development


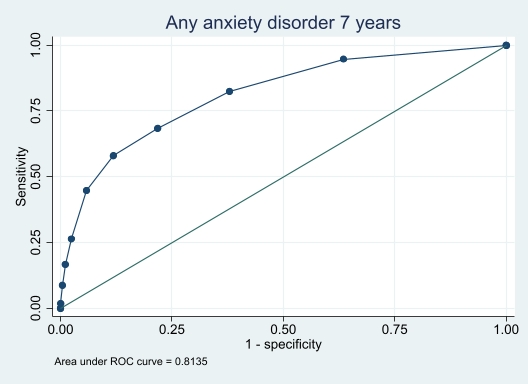

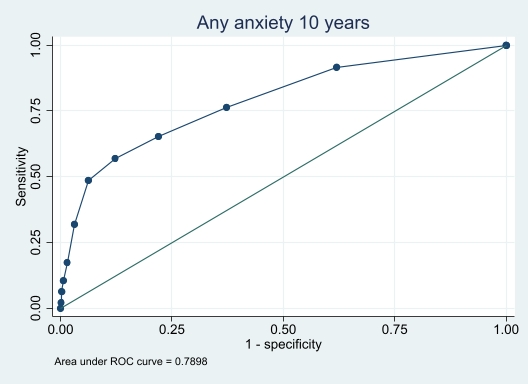

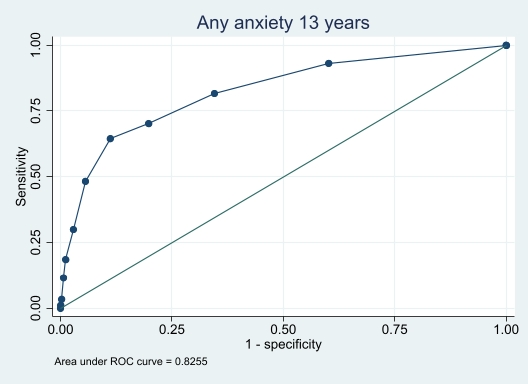

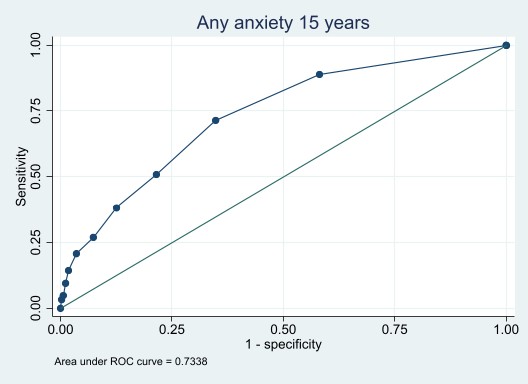

Supplement: S1 Fig — (DOCX) [file pone.0288882.s001.docx]
