# Supplementary material for: Validation of the Strengths and Difficulties Questionnaire (SDQ) emotional subscale in assessing depression and anxiety across development
Source: PLoS One. 2023 Jul 19;18(7):e0288882. doi: 10.1371/journal.pone.0288882 (PMC10355443; doi:10.1371/journal.pone.0288882)
Supplement: S1 Table — (DOCX) [file pone.0288882.s003.docx]

**material**

**Validation of the Strengths and Difficulties Questionnaire (SDQ) emotional subscale in assessing depression and anxiety across development**

Armitage, J. M ^a,b^., Tseliou, F ^a,b^., Riglin, L ^a,b^., Dennison, C, A ^a,b^., Eyre, O ^a,b^., Bevan Jones, R^a,b^., Rice, F^a,b^., Thapar, A. K ^a,b^., Thapar, A ^a,b^., & Collishaw, S ^a,b^

^a^ Wolfson Centre for Young People’s Mental Health, Cardiff University, Wales, United Kingdom

^b^ Division of Psychological Medicine and Clinical Neurosciences, Centre for Neuropsychiatric Genetics and Genomics, Cardiff University, Wales, United Kingdom

- Table S1: Descriptives of samples included
- Table S2: Descriptives of Strengths and Difficulties Questionnaire by sex
- Table S3: Variables included in multiple imputation
- Table S4: Discrimination of those with versus without DAWBA diagnoses for the emotional subscale and depressive and worry items using imputed dataset (n=9,241)
- Table S5: Sensitivity and specificity of the emotional subscale cutoff-points across development compared against Major Depressive Disorder Diagnoses
- Table S6: Sensitivity and specificity of the emotional subscale cutoff-points across development compared against Generalised Anxiety Disorder Diagnoses
- Table S7: Sensitivity and specificity of the emotional subscale cutoff-points across development compared against any anxiety disorder diagnoses
- Table S8: Accuracy of identifying those meeting diagnostic criteria by optimal SDQ subscale cut-point
- Table S9: Discrimination of those with versus without DAWBA diagnoses for the depressive item by sex
- Table S10: Discrimination of those with versus without DAWBA diagnoses for the worry item by sex
- Table S11: Comorbidity of DAWBA Depressive and Anxiety disorders by sex
- **Fig S1:** ROC analyses for emotional subscale predicting any anxiety diagnosis across development
- **Fig S2:** ROC analyses for emotional subscale predicting Attention Deficit Hyperactivity Disorder or any behavioural diagnosis across development

| **Table S1: Descriptives of samples included** | | | | | | | | | | | | | | | | |
| --- | --- | --- | --- | --- | --- | --- | --- | --- | --- | --- | --- | --- | --- | --- | --- | --- |
| **Strengths and Difficulties Questionnaire (SDQ)** | | | | | | | | **Development and Well-Being Assessment (DAWBA) Diagnoses** | | | | | | | | |
| **Age** | **Emotional subscale**  **(range 0-10)** | | | **Depressive item**  **(range 0-2)** | | **Worry item**  **(range 0-2)** | | **Age** | **Major Depressive disorder** | | **Generalised Anxiety Disorder** | | **Any anxiety disorder** | | **Attention Deficit Hyperactivity Disorder (ADHD) or any behavioural disorder*** | |
|  | N | Mean (SD) | α | N | Mean (SD) | N | Mean (SD) |  | N | With disorder (%) | N | With disorder (%) | N | With disorder (%) | N | With disorder (%) |
| 7  years | 8,312 | 1.51 (1.67) | 0.63 | 8,298 | 0.17 (0.41) | 8,256 | 0.29 (0.52) | 7 years | 7987 | 52 (0.7%) | 8,098 | 17 (0.2%) | 8,041 | 138 (1.7%) | 8,102 | 293 (3.6%) |
| 10 years | 7,956 | 1.52 (1.77) | 0.68 | 7,905 | 0.18 (0.42) | 7,833 | 0.30 (0.52) | 10 years | 7,560 | 74 (1.0%) | 7,674 | 33 (0.4%) | 8,063 | 160 (2.2%) | 7,558 | 245 (3.3%) |
| 13 years | 6,970 | 1.44 (1.72) | 0.67 | 6,936 | 0.18 (0.43) | 6,914 | 0.29 (0.52) | 13 years | 6,871 | 58 (0.8%) | 6,969 | 31 (0.4%) | 6,401 | 104 (1.6%) | 6,638 | 240 (3.5%) |
| 16 years | 5,590 | 1.50 (1.86) | 0.71 | 5,558 | 0.19 (0.46) | 5,504 | 0.40 (0.60) | 15 years  (self) | 5,293 | 86 (1.6%) | 5,289 | 38 (0.7%) | 5,275 | 101 (1.9%) | 4,533 | 186 (4.0%) |
| 25 years | 4,393 | 1.90 (2.23) | 0.77 | 4,387 | 0.26 (0.55) | 4,319 | 0.54 (0.68) | 25 years  (self) | 4,074 | 378 (9.3%) | - | - | - | - | - | - |
| 25 years  (self) | 4,309 | 3.44 (2.49) | 0.75 | 4,305 | 0.49 (0.66) | 4,295 | 1.10 (0.77) | - | - | - | - | - | - | - | - | - |
| Note: α = Cronbach’s alpha. *Any behavioural disorder includes Conduct Disorder (CD) and Oppositional Defiant Disorder (ODD).  Sample sizes for the individual items are lower than the total subscale as this used a mean imputation procedure for those missing ≤2 items. All SDQ assessments are based on parent-reports unless stated otherwise. DAWBA diagnoses at ages 7, 10 and 13 years are based on parent-reports, while diagnoses at 15 and 25 are based on self-reports. At 7 years, the mean age during assessment of the SDQ was 81 months, and 91 months for the DAWBA diagnoses. At age 10, the mean age of assessment for the SDQ was 115 months, and 128 months for the DAWBA. At 13 years mean ages were 157 months and 166 months respectively, and at 15/16 years, the SDQ was parent-rated at 198 months, while the DAWBA diagnoses were self-reported at 185 months. | | | | | | | | | | | | | | | | |
