# Supplementary material for: Validation of the Strengths and Difficulties Questionnaire (SDQ) emotional subscale in assessing depression and anxiety across development
Source: PLoS One. 2023 Jul 19;18(7):e0288882. doi: 10.1371/journal.pone.0288882 (PMC10355443; doi:10.1371/journal.pone.0288882)
Supplement: S2 Table — (DOCX) [file pone.0288882.s004.docx]

| **Table S2: Descriptives of Strengths and Difficulties Questionnaire by sex** | | | | | | | | | | | | | | | |
| --- | --- | --- | --- | --- | --- | --- | --- | --- | --- | --- | --- | --- | --- | --- | --- |
|  | **Emotional subscale (range 0-10)** | | | | | **Depressive item (range 0-2)** | | | | | **Worry item (range 0-2)** | | | | |
| **Age** | **Males** | | **Females** | | **Diff *** | **Males** | | **Females** | | **Diff *** | **Males** | | **Females** | | **Diff *** |
|  | N | Mean (SD) | N | Mean  (SD) | Estimate  (95% CI) | N | Mean (SD) | N | Mean  (SD) | Estimate  (95% CI) | N | Mean (SD) | N | Mean  (SD) | Estimate  (95% CI) |
| 7 years | 4,265 | 1.43 (1.66) | 4,047 | 1.58 (1.68) | **0.15**  (0.08, 0.22) | 4,258 | 0.16 (0.41) | 4,040 | 0.18 (0.41) | 0.02  (-0.004, 0.03) | 4,231 | 0.29 (0.53) | 4,025 | 0.29 (0.51) | 0.00  (-0.03, 0.02) |
| 10 years | 4,015 | 1.39 (1.71) | 3,941 | 1.66 (1.82) | **0.27**  (0.20, 0.35) | 3,986 | 0.16 (0.40) | 3,919 | 0.20 (0.44) | **0.04**  (0.02, 0.05) | 3,957 | 0.29 (0.52) | 3,876 | 0.30 (0.52) | 0.01  (-0.01, 0.04) |
| 13 years | 3,471 | 1.24 (1.61) | 3,499 | 1.63 (1.79) | **0.39**  (0.31, 0.47) | 3,465 | 0.15 (0.40) | 3,480 | 0.20 (0.46) | **0.05**  (0.03, 0.07) | 3,443 | 0.26 (0.50) | 3,471 | 0.31 (0.53) | **0.04**  (0.02, 0.07) |
| 16 years | 2,676 | 1.08 (1.55) | 2,878 | 1.87 (2.03) | **0.79**  (0.69, 0.88) | 2,676 | 0.11 (0.37) | 2,882 | 0.26 (0.52) | **0.15**  (0.12, 0.17) | 2,641 | 0.32 (0.56) | 2,863 | 0.46 (0.63) | **0.14**  (0.11, 0.17) |
| 25 years | 2,042 | 1.50 (1.94) | 2,351 | 2.25 (2.40) | **0.74**  (0.61, 0.87) | 2,041 | 0.20 (0.49) | 2,346 | 0.32 (0.59) | **0.12**  (0.08, 0.15) | 2,001 | 0.46 (0.64) | 2,318 | 0.60 (0.71) | **0.14**  (0.10, 0.18) |
| 25 years  (self) | 1,455 | 2.62 (2.21) | 2,849 | 3.87 (2.52) | **1.25**  (1.10, 1.40) | 1,454 | 0.40 (0.64) | 2,846 | 0.53 (0.67) | **0.13**  (0.09, 0.17) | 1,451 | 0.85 (0.76) | 2,839 | 1.23 (0.74) | **0.38**  (0.33, 0.43) |
| Note: *Difference represents the degree to which females score more highly than males at that age point. Those in bold are statistically different.  Sample sizes for the individual items are lower than the total subscale as this used a mean imputation procedure for those missing <2 items. All SDQ assessments are based on parent-reports unless stated otherwise. At 7 years, the mean age during assessment of the SDQ was 81 months, and 91 months for the DAWBA diagnoses. At age 10, the mean age of assessment for the SDQ was 115 months, and 128 months for the DAWBA. At 13 years the SDQ and DAWBA were assessed at mean ages 157 months and 166 months respectively, and at 15/16 years, the SDQ was assessed at 198 months, while the DAWBA diagnoses were self-reported at 185 months. | | | | | | | | | | | | | | | |
