# Supplementary material for: Validation of the Strengths and Difficulties Questionnaire (SDQ) emotional subscale in assessing depression and anxiety across development
Source: PLoS One. 2023 Jul 19;18(7):e0288882. doi: 10.1371/journal.pone.0288882 (PMC10355443; doi:10.1371/journal.pone.0288882)
Supplement: S3 Table — (DOCX) [file pone.0288882.s005.docx]

| **Table S3: Variables included in multiple imputation** | | | | |
| --- | --- | --- | --- | --- |
| **Variable** | **Age at assesment** | **Respondent** | **Item** | **Item response** |
| SDQ emotional problems subscale items | 7, 10, 13, 16, and 25 years | Mother | Strengths and Difficulties Questionnaire (SDQ) | ‘Not true’/‘Somewhat true’/‘Certainly true’ |
| Participant MDD, GAD, any anxiety, and any ADHD or behavioural diagnosis | 7, 10, 13, 15, and 25 years  25 years | Mother  Self | Development and Well-Being Assessment (DAWBA) | With disorder/Without disorder |
| Family ethnicity | 32 weeks gestation | Mother | “How would you describe the race or ethnic group of yourself/your partner?” | 8 response options. |
| Mother’s age at first pregnancy | 18 weeks gestation | Mother | “How old were you when you became pregnant for the very first time?” | Age in years. |
| Mother marital status | 32 weeks gestation | Mother | “What is your present marital status? | 6 response options including “Married” and “Separated” |
| Mother educational qualifications | 32 weeks gestation | Mother | “What educational qualifications do you/your partner have?” | List of qualifications, respondent required to tick all that apply |
| Mother home ownership status | 8 months | Mother | “Do you currently live in..” | 6 response options including “Mortgaged” and “Rented from private landlord” |
| Mother economic status | 32 weeks gestation | Mother | “What is the present employment situation of yourself?” | 11 response options including “Working for an employer full time”, “Self-employed”, “In full time education” and “Looking after home/family” |
| Mother smoking during pregnancy | 8 weeks | Mother | Did you smoke regularly in the last 2 months of pregnancy and since having the baby? | Options include no/ yes (cigarettes/pipe/cigar/other) |
| Mother mental health during pregnancy | 8 months | Mother | Edinburgh Post-natal Depression Score | 10 item questionnaire rated. Those scoring 13 or above are considered to be suffering from depressive disorder |
