# Supplementary material for: Validation of the Strengths and Difficulties Questionnaire (SDQ) emotional subscale in assessing depression and anxiety across development
Source: PLoS One. 2023 Jul 19;18(7):e0288882. doi: 10.1371/journal.pone.0288882 (PMC10355443; doi:10.1371/journal.pone.0288882)
Supplement: S4 Table — (DOCX) [file pone.0288882.s006.docx]

| **Table S4: Discrimination of those with versus without DAWBA diagnoses for the emotional subscale using the imputed dataset (n=9,241)** | | | | |
| --- | --- | --- | --- | --- |
| **Age (years)** | **Emotional subscale**  **AUC (95% CI)** | | | |
|  |  | | | |
|  | **Major Depressive Disorder** | **Generalised Anxiety Disorder** | **Any anxiety disorder** | **Attention Deficit Hyperactivity Disorder (ADHD) or any behavioural disorder*** |
| 7 years | 0.77 (0.70, 0.84) | 0.89 (0.78, 0.99) | 0.81 (0.77, 0.85) | 0.61 (0.58, 0.65) |
| 10 years | 0.76 (0.70, 0.82) | 0.86 (0.78, 0.94) | 0.78 (0.74, 0.82) | 0.65 (0.61, 0.69) |
| 13 years | 0.82 (0.76, 0.88) | 0.91 (0.85, 0.97) | 0.82 (0.77, 0.87) | 0.64 (0.60, 0.68) |
| 15/16 years | 0.68 (0.61, 0.74) | 0.74 (0.65, 0.84) | 0.71 (0.66, 0.77) | 0.70 (0.66, 0.74) |
| 25 years | 0.74 (0.70, 0.77) | - | - | **-** |
| 25 years (self) | 0.84 (0.82, 0.86) | - | - | - |
| Note: Any behavioural disorder includes Conduct Disorder (CD) and Oppositional Defiant Disorder (ODD). SDQ assessments are based on the concurrent age of the diagnosis, however there is a gap between assessments. All SDQ assessments are based on parent-reports unless stated otherwise. Diagnoses at ages 7, 10 and 13 years are based on parent-reports, while diagnoses at 15 and 25 years are based on self-reports. | | | | |
