# Supplementary material for: Validation of the Strengths and Difficulties Questionnaire (SDQ) emotional subscale in assessing depression and anxiety across development
Source: PLoS One. 2023 Jul 19;18(7):e0288882. doi: 10.1371/journal.pone.0288882 (PMC10355443; doi:10.1371/journal.pone.0288882)
Supplement: S5 Table — (DOCX) [file pone.0288882.s007.docx]

| **Table S5: Sensitivity and specificity of the emotional subscale cutoff-points across development compared against Major Depressive Disorder Diagnoses** | | | | | | | | | | | | |
| --- | --- | --- | --- | --- | --- | --- | --- | --- | --- | --- | --- | --- |
| Cut-point | Major Depressive Disorder at 7 years | | Major Depressive Disorder at 10 years | | Major Depressive Disorder at 13 years | | Major Depressive Disorder at 15/16 years | | Major Depressive Disorder at 25 years (based on parent-rated SDQ) | | Major Depressive Disorder at 25 years (based on self-rated SDQ) | |
|  | Sensitivity | Specificity | Sensitivity | Specificity | Sensitivity | Specificity | Sensitivity | Specificity | Sensitivity | Specificity | Sensitivity | Specificity |
| ≥ 1 | 90.48% | 36.14% | 90.63% | 37.56% | 95.74% | 39.50% | 79.25% | 41.72% | 86.60% | 39.15% | 100.00% | 12.88% |
| ≥ 2 | 76.19% | 61.69% | **81.25%**  **PPV=2%** | **62.33%**  **NPV=>99%** | 87.23% | 65.13% | 66.04% | 65.14% | 73.71% | 56.98% | 98.94% | 28.77% |
| ≥ 3 | **61.90%**  **PPV=2%** | **77.73%**  **NPV=>99%** | 57.81% | 77.48% | **78.72%**  **PPV=3%** | **79.95%**  **NPV=>99%** | **52.83%**  **PPV=3%** | **78.57%**  **NPV=>99%** | **59.28%**  **PPV=15%** | **73.06%**  **NPV=96%** | 94.97% | 45.74% |
| ≥ 4 | 50.00% | 87.72% | 46.88% | 87.14% | 59.57% | 88.51% | 26.42% | 87.17% | 47.42% | 83.24% | 90.21% | 60.46% |
| ≥ 5 | 33.33%  PPV=3% | 93.79%  NPV=99% | 34.38%  PPV=5% | 93.20%  NPV=99% | 40.43%  PPV=5% | 94.07%  NPV=99% | 15.09%  PPV=3% | 92.49%  NPV=99% | 38.14%  PPV=24% | 89.90%  NPV=95% | **83.07%**  **PPV=24%** | **72.37%**  **NPV=98%** |
| ≥ 6 | 23.81% | 97.38% | 21.88% | 96.49% | 27.66% | 96.90% | 11.32% | 96.33% | 31.44% | 93.73% | 69.31% | 83.41% |
| ≥ 7 | 14.29% | 98.79% | 7.81% | 98.30% | 17.02% | 98.66% | 7.55% | 98.17% | 23.71% | 95.96% | 52.91% | 90.66% |
| ≥ 8 | 9.52% | 99.52% | 4.69% | 99.26% | 10.64% | 99.25% | 5.66% | 98.79% | 17.53% | 97.46% | 34.92% | 95.48% |
| ≥ 9 | 2.38% | 99.90% | 3.13% | 99.61% | 2.13% | 99.68% | 3.77% | 99.41% | 12.89% | 98.58% | 18.78% | 98.21% |
| ≥ 10 | 0.00% | 99.99% | 1.56% | 99.87% | 2.13% | 99.92% | 3.77% | 99.74% | 8.25% | 99.57% | 9.26% | 99.49% |
| Note: PPV=Positive predictive values. NPV = Negative predictive values.  Sensitivity and specificity estimates of the SDQ emotional subscale are based on assessments at the concurrent age of the depression diagnoses (although note there is a slight age gap between SDQ and diagnosis assessments). All but the SDQ assessment at 25 years are based on parent-reports. Depression diagnoses at ages 7, 10, and 13 years are based on parent-reports, while diagnoses at 15 and 25 years are based on self-reports. Scores on the SDQ emotional subscale of 5 and above have been suggested to capture those with ‘high’ problems (see sdqinfo.org). Estimates in bold represent the optimum cut-point according to the balance of sensitivity and specificity. | | | | | | | | | | | | |
