# Supplementary material for: Validation of the Strengths and Difficulties Questionnaire (SDQ) emotional subscale in assessing depression and anxiety across development
Source: PLoS One. 2023 Jul 19;18(7):e0288882. doi: 10.1371/journal.pone.0288882 (PMC10355443; doi:10.1371/journal.pone.0288882)
Supplement: S6 Table — (DOCX) [file pone.0288882.s008.docx]

| **Table S6: Sensitivity and specificity of the emotional subscale cutoff-points across development compared against Generalised Anxiety Disorder Diagnoses** | | | | | | | | |
| --- | --- | --- | --- | --- | --- | --- | --- | --- |
| Cut-point | Generalised Anxiety Disorder at 7 years | | Generalised Anxiety Disorder at 10 years | | Generalised Anxiety Disorder at 13 years | | Generalised Anxiety Disorder at 15/16 years | |
|  | Sensitivity | Specificity | Sensitivity | Specificity | Sensitivity | Specificity | Sensitivity | Specificity |
| ≥ 1 | 100% | 36.14% | 96.67% | 37.59% | 100.00% | 39.49% | 100.00% | 41.68% |
| ≥ 2 | 92.31% | 61.55% | 83.33% | 62.11% | 96.00% | 65.02% | **81.82%**  **PPV=1%** | **65.01%**  **NPV=>99%** |
| ≥ 3 | 84.62% | 77.60% | 80.00% | 77.36% | 96.00% | 79.78% | 68.18% | 78.44% |
| ≥ 4 | 76.92% | 87.61% | **76.67%**  **PPV=3%** | **87.11%**  **NPV=>99%** | **88.00%**  **PPV=3%** | **88.45%**  **NPV=>99%** | 40.91% | 87.16% |
| ≥ 5 | **76.92%**  **PPV=2%** | **93.73%**  **NPV=>99%** | 70.00%  PPV=4% | 93.19%  NPV=>99% | 60.00%  PPV=4% | 94.05%  NPV=>99% | 18.18%  PPV=1% | 92.46%  NPV=>99% |
| ≥ 6 | 46.15% | 97.34% | 53.33% | 96.54% | 48.00% | 96.95% | 13.64% | 96.28% |
| ≥ 7 | 38.46% | 98.79% | 30.00% | 98.35% | 28.00% | 98.66% | 13.64% | 98.15% |
| ≥ 8 | 30.77% | 99.53% | 20.00% | 99.28% | 16.00% | 99.24% | 13.64% | 98.80% |
| ≥ 9 | 0.00% | 99.89% | 10.00% | 99.62% | 8.00% | 99.70% | 9.09% | 99.41% |
| ≥ 10 | 0.00% | 99.99% | 6.67% | 99.88% | 4.00% | 99.92% | 4.55% | 99.72% |
| Note: PPV=Positive predictive values. NPV = Negative predictive values.  Sensitivity and specificity estimates of the SDQ emotional subscale are based on assessments at the concurrent age of the GAD diagnoses (although note there is a slight age gap between SDQ and diagnosis assessments). All but the SDQ assessment at 25 years are based on parent-reports. GAD diagnoses at ages 7, 10, and 13 years are based on parent-reports, while diagnoses at 15 years are based on self-reports. Scores on the SDQ emotional subscale of 5 and above have been suggested to capture those with ‘high’ problems (see sdqinfo.org). | | | | | | | | |
