# Supplementary material for: Validation of the Strengths and Difficulties Questionnaire (SDQ) emotional subscale in assessing depression and anxiety across development
Source: PLoS One. 2023 Jul 19;18(7):e0288882. doi: 10.1371/journal.pone.0288882 (PMC10355443; doi:10.1371/journal.pone.0288882)
Supplement: S7 Table — (DOCX) [file pone.0288882.s009.docx]

| **Table S7: Sensitivity and specificity of the emotional subscale cutoff-points across development compared against any anxiety disorder diagnoses** | | | | | | | | |
| --- | --- | --- | --- | --- | --- | --- | --- | --- |
| Cut-point | Any Anxiety Disorder at 7 years | | Any Anxiety Disorder at 10 years | | Any Anxiety Disorder at 13 years | | Any Anxiety Disorder at 15/16 years | |
|  | Sensitivity | Specificity | Sensitivity | Specificity | Sensitivity | Specificity | Sensitivity | Specificity |
| ≥1 | 94.69% | 36.50% | 91.49% | 37.63% | 94.12% | 39.12% | 88.89% | 41.88% |
| ≥ 2 | 82.30% | 62.14% | 75.89% | 62.28% | 82.35% | 64.64% | **71.43%**  **PPV=3%** | **65.14%**  **NPV=>99%** |
| ≥ 3 | **68.14%**  **PPV=5%** | **78.17%**  **NPV=99%** | 64.54% | 77.57% | 70.59% | 79.56% | 50.79% | 78.52% |
| ≥ 4 | 57.52% | 88.18% | **56.74%**  **PPV=9%** | **87.49%**  **NPV=>99%** | **64.71%**  **PPV=8%** | **88.57%**  **NPV=>99%** | 38.10% | 87.42% |
| ≥ 5 | 44.25% | 94.21% | 48.23% | 93.68% | 49.41% | 94.25% | 26.98% | 92.68% |
| ≥ 6 | 26.55% | 97.63% | 32.62% | 96.85% | 30.59% | 97.08% | 20.63% | 96.47% |
| ≥ 7 | 16.81% | 98.98% | 17.73% | 98.54% | 18.82% | 98.84% | 14.29% | 98.22% |
| ≥ 8 | 8.85% | 99.62% | 10.64% | 99.40% | 11.76% | 99.38% | 9.52% | 98.84% |
| ≥ 9 | 1.77% | 99.91% | 6.38% | 99.69% | 3.53% | 99.75% | 4.76% | 99.43% |
| ≥ 10 | 0.00% | 99.99% | 2.13% | 99.89% | 1.18% | 99.93% | 3.17% | 99.74% |
| Note: PPV=Positive predictive values. NPV = Negative predictive values.  Sensitivity and specificity estimates of the SDQ emotional subscale are based on assessments at the concurrent age of the anxiety diagnosis (at ages 7, 10, 13, 15/16, and 25 years – although note there is a slight age gap between SDQ and diagnosis assessments). All but the SDQ assessment at 25 years are based on parent-reports. Any anxiety diagnosis at ages 7, 10 and 13 years are based on parent-reports, while diagnoses at 15 years are based on self-reports. Scores on the SDQ emotional subscale of 5 and above have been suggested to capture those with ‘high’ problems (see sdqinfo.org). | | | | | | | | |
