# Supplementary material for: Validation of the Strengths and Difficulties Questionnaire (SDQ) emotional subscale in assessing depression and anxiety across development
Source: PLoS One. 2023 Jul 19;18(7):e0288882. doi: 10.1371/journal.pone.0288882 (PMC10355443; doi:10.1371/journal.pone.0288882)
Supplement: S8 Table — (DOCX) [file pone.0288882.s010.docx]

| **Table S8: Accuracy of identifying those meeting diagnostic criteria by optimal SDQ subscale cut-point** | | | | | | | | |
| --- | --- | --- | --- | --- | --- | --- | --- | --- |
| **Major Depressive Disorder** | | | **Generalised anxiety disorder** | | | **Any anxiety disorder** | | |
| Optimal cut-point | Met diagnostic criteria | Did not meet diagnostic criteria | Optimal cut-point | Met diagnostic criteria | Did not meet diagnostic criteria | Optimal cut-point | Met diagnostic criteria | Did not meet diagnostic criteria |
| **7 years** |  |  | **7 years** |  |  | **7 years** |  |  |
| (cut-off ≥ 3) | 26 (62%) | 1,578 (29%) | (cut-off ≥ 5) | 10 (77%) | 452 (6%) | (cut-off ≥ 3) | 78 (68%) | 1,541 (22%) |
| (cut-off < 3) | 16 (38%) | 5,509 (71%) | (cut-off < 5) | 3 (23%) | 6,753 (94%) | (cut-off < 3) | 36 (32%) | 5,517 (78%) |
| **10 years** |  |  | **10 years** |  |  | **10 years** |  |  |
| (cut-off ≥ 2) | 52 (81%) | 2,531 (38%) | (cut-off ≥ 4) | 23 (77%) | 882 (13%) | (cut-off ≥ 4) | 82 (57%) | 807 (13%) |
| (cut-off < 2) | 12 (19%) | 4,186 (62%) | (cut-off < 4) | 7 (23%) | 5,961 (87%) | (cut-off < 4) | 62 (43%) | 5,645 (87%) |
| **13 years** |  |  | **13 years** |  |  | **13 years** |  |  |
| (cut-off ≥ 3) | 37 (79%) | 1,197 (20%) | (cut-off ≥ 4) | 22 (88%) | 701 (12%) | (cut-off ≥ 4) | 56 (64%) | 630 (11%) |
| (cut-off < 3) | 10 (21%) | 4,773 (80%) | (cut-off < 4) | 3 (12%) | 5,366 (88%) | (cut-off < 4) | 31 (36%) | 4,884 (89%) |
| **15/16 years** |  |  | **15/16 years** |  |  | **15/16 years** |  |  |
| (cut-off ≥ 3) | 28 (52%) | 841 (22%) | (cut-off ≥ 2) | 18 (82%) | 1,381 (35%) | (cut-off ≥ 2) | 46 (72%) | 1,352 (35%) |
| (cut-off < 3) | 26 (48%) | 3,054 (78%) | (cut-off < 2) | 4 (18%) | 2,541 (65%) | (cut-off < 2) | 18 (28%) | 2,526 (65%) |
| **25 years** |  |  | - |  |  | - |  |  |
| (cut-off ≥ 3) | 115 (59%) | 627 (27%) | - |  |  | - |  |  |
| (cut-off < 3) | 79 (41%) | 1,700 (73%) | - |  |  | - |  |  |
| **25 years (self)** |  |  | - |  |  | - |  |  |
| (cut-off ≥ 5) | 314 (83%) | 1,021 (28%) | - |  |  | - |  |  |
| (cut-off < 5) | 64 (17%) | 2,674 (72%) | - |  |  | - |  |  |
| Note: SDQ assessments are based on the concurrent age of the diagnosis, however there is some gap between assessments. All SDQ assessments are based on parent-reports unless stated otherwise. Diagnoses at ages 7, 10 and 13 years are based on parent-reports, while diagnoses at 15 and 25 years are based on self-reports. | | | | | | | | |
