# Supplementary material for: Validation of the Strengths and Difficulties Questionnaire (SDQ) emotional subscale in assessing depression and anxiety across development
Source: PLoS One. 2023 Jul 19;18(7):e0288882. doi: 10.1371/journal.pone.0288882 (PMC10355443; doi:10.1371/journal.pone.0288882)
Supplement: S9 Table — (DOCX) [file pone.0288882.s011.docx]

| **Table S9: Discrimination of those with versus without DAWBA diagnoses for the depressive item by sex** | | | | | | | | | | | | |
| --- | --- | --- | --- | --- | --- | --- | --- | --- | --- | --- | --- | --- |
|  | **Major Depressive Disorder** | | | **Generalised Anxiety Disorder** | | | **Any anxiety disorder** | | | **Attention Deficit Hyperactivity Disorder (ADHD) or any behavioural disorder** | | |
| **Age** | **Males** | **Females** | **Diff** | **Males** | **Females** | **Diff** | **Males** | **Females** | **Diff** | **Males** | **Females** | **Diff** |
|  | AUC  (95% CI) | AUC  (95% CI) | χ^2^_(1)_, p-value | AUC  (95% CI) | AUC  (95% CI) | χ^2^_(1)_, p-value | AUC  (95% CI) | AUC  (95% CI) | χ^2^_(1)_, p-value | AUC  (95% CI) | AUC  (95% CI) | χ^2^_(1)_, p-value |
| 7 years | 0.76  (0.66, 0.86) | 0.61  (0.48, 0.73) | 3.71,  **<0.05** | 0.80  (0.64, 0.96) | 0.75  (0.43, 1.00) | 0.08,  0.77 | 0.66  (0.59, 0.72) | 0.59  (0.52, 0.66) | 1.89,  0.17 | 0.60  (0.57, 0.64) | 0.62  (0.56, 0.69) | 0.35,  0.55 |
| 10 years | 0.67  (0.58, 0.76) | 0.65  (0.56, 0.75) | 0.08,  0.78 | 0.78  (0.66, 0.90) | 0.71  (0.54, 0.88) | 0.36,  0.55 | 0.70  (0.64, 0.76) | 0.64  (0.58, 0.70) | 2.05,  0.15 | 0.62  (0.58, 0.66) | 0.66  (0.59, 0.72) | 0.75,  0.39 |
| 13 years | 0.75  (0.65, 0.85) | 0.76  (0.66, 0.86) | 0.00,  0.95 | 0.76  (0.63, 0.90) | 0.88  (0.78, 0.97) | 1.91,  0.17 | 0.70  (0.63, 0.79) | 0.68  (0.60, 0.75) | 0.31,  0.58 | 0.60  (0.55, 0.64) | 0.62  (0.57, 0.68) | 0.49,  0.49 |
| 15/16 years | 0.55  (0.45, 0.66) | 0.62  (0.53, 0.70) | 0.86,  0.35 | 0.45  (0.45, 0.46) | 0.75  (0.65, 0.86) | 29.66,  **<0.001** | 0.45  (0.45, 0.46) | 0.67  (0.60, 0.74) | 35.99  **<0.001** | 0.60  (0.54, 0.66) | 0.71  (0.64, 0.78) | 5.35,  **<0.05** |
| 25 years | 0.68  (0.60, 0.76) | 0.68  (0.64, 0.72) | 0.00,  0.97 | - | - | - | - | - | - | - | - | - |
| 25 years  (self) | 0.89  (0.85, 0.92) | 0.85  (0.83, 0.87) | 3.20,  0.07 | - | - | - | - | - | - | - | - | - |
| Note: *Any behavioural disorder includes Conduct Disorder (CD) and Oppositional Defiant Disorder (ODD). All DAWBA diagnoses at ages 7, 10 and 13 years are based on parent-reports, while diagnoses at 15 and 25 are based on self-reports. | | | | | | | | | | | | |
