# Supplementary material for: Validation of the Strengths and Difficulties Questionnaire (SDQ) emotional subscale in assessing depression and anxiety across development
Source: PLoS One. 2023 Jul 19;18(7):e0288882. doi: 10.1371/journal.pone.0288882 (PMC10355443; doi:10.1371/journal.pone.0288882)
Supplement: S10 Table — (DOCX) [file pone.0288882.s012.docx]

| **Table S10: Discrimination of those with versus without DAWBA diagnoses for the worry item by sex** | | | | | | | | | | | | |
| --- | --- | --- | --- | --- | --- | --- | --- | --- | --- | --- | --- | --- |
|  | **Major Depressive Disorder** | | | **Generalised Anxiety Disorder** | | | **Any anxiety disorder** | | | **Attention Deficit Hyperactivity Disorder (ADHD) or any** **behavioural disorder*** | | |
| **Age** | **Males** | **Females** | **Diff** | **Males** | **Females** | **Diff** | **Males** | **Females** | **Diff** | **Males** | **Females** | **Diff** |
|  | AUC  (95% CI) | AUC  (95% CI) | χ^2^_(1)_, p-value | AUC  (95% CI) | AUC  (95% CI) | χ^2^_(1)_, p-value | AUC  (95% CI) | AUC  (95% CI) | χ^2^_(1)_, p-value | AUC  (95% CI) | AUC  (95% CI) | χ^2^_(1)_, p-value |
| 7 years | 0.77  (0.67, 0.86) | 0.67  (0.53, 0.80) | 1.35,  0.25 | 0.80  (0.65, 0.94) | 0.94  (0.86, 1.00) | 2.93,  0.09 | 0.71  (0.64, 0.77) | 0.71  (0.64, 0.79) | 0.01, 0.94 | 0.57  (0.53, 0.60) | 0.59  (0.53, 0.65) | 0.45,  0.50 |
| 10 years | 0.75  (0.67, 0.83) | 0.75  (0.67, 0.84) | 0.01,  0.93 | 0.83  (0.74, 0.93) | 0.79  (0.63, 0.96) | 0.19,  0.67 | 0.75  (0.69, 0.81) | 0.66  (0.60, 0.73) | 3.40,  0.07 | 0.61  (0.57, 0.66) | 0.61  (0.55, 0.68) | 0.00,  0.98 |
| 13 years | 0.70  (0.60, 0.81) | 0.77  (0.67, 0.88) | 0.91,  0.34 | 0.85  (0.75, 0.96) | 0.89  (0.85, 0.92) | 0.32,  0.57 | 0.76  (0.68, 0.83) | 0.74  (0.66, 0.81) | 0.10,  0.75 | 0.60  (0.56, 0.65) | 0.63  (0.57, 0.69) | 0.34,  0.56 |
| 15/16 years | 0.70  (0.56, 0.84) | 0.63  (0.54, 0.72) | 0.81,  0.37 | 0.85  (0.83, 0.86) | 0.68  (0.56, 0.80) | 7.70,  **<0.01** | 0.51  (0.36, 0.66) | 0.64  (0.56, 0.71) | 2.38, 0.12 | 0.63  (0.56, 0.69) | 0.66  (0.58, 0.73) | 0.48,  0.49 |
| 25 years (parent) | 0.64  (0.56, 0.72) | 0.67  (0.62, 0.71) | 0.32, 0.57 | - | - | - | - | - | - | - | - | - |
| 25 years (self) | 0.77  (0.73, 0.82) | 0.71  (0.69, 0.74) | 5.27,  **<0.05** | - | - | - | - | - | - | - | - | - |
| Note: *Any behavioural disorder includes Conduct Disorder (CD) and Oppositional Defiant Disorder (ODD). All DAWBA diagnoses at ages 7, 10 and 13 years are based on parent-reports, while diagnoses at 15 and 25 are based on self-reports. | | | | | | | | | | | | |
