# Supplementary material for: Validation of the Strengths and Difficulties Questionnaire (SDQ) emotional subscale in assessing depression and anxiety across development
Source: PLoS One. 2023 Jul 19;18(7):e0288882. doi: 10.1371/journal.pone.0288882 (PMC10355443; doi:10.1371/journal.pone.0288882)
Supplement: S11 Table — (DOCX) [file pone.0288882.s013.docx]

| **Table S11: Comorbidity of DAWBA Depressive and Anxiety disorders by sex** | | | | | | | | | | |
| --- | --- | --- | --- | --- | --- | --- | --- | --- | --- | --- |
| Age | **Major Depressive disorder** | | | | **Generalised Anxiety Disorder** | | | **Any anxiety disorder** | | |
|  | N | With disorder (%) | Diagnosed and have GAD (%) | Diagnosed and have any anxiety disorder (%) | N | With disorder (%) | Diagnosed and have MDD (%) | N | With disorder (%) | Diagnosed and have MDD (%) |
| **All** |  |  |  |  |  |  |  |  |  |  |
| 7 years | 7,987 | 52 (0.7%) | 8 (15.4%) | 18 (34.6%) | 8,098 | 17 (0.2%) | 8 (47.1%) | 8,041 | 138 (1.7%) | 17 (13.0%) |
| 10 years | 7,560 | 74 (1.0%) | 12 (16.2%) | 21 (28.4%) | 7,674 | 33 (0.4%) | 12 (36.4%) | 8,063 | 160 (2.2%) | 21 (13.0%) |
| 13 years | 6,871 | 58 (0.8%) | 12 (20.7%) | 19 (32.8%) | 6,969 | 31 (0.4%) | 12 (38.7%) | 6,401 | 104 (1.6%) | 19 (18.3%) |
| 15 years | 5,293 | 86 (1.6%) | 13 (15.1%) | 22 (25.6%) | 5,289 | 38 (0.7%) | 13 (34.2%) | 5,275 | 101 (1.9%) | 22 (21.8%) |
| **Males** |  |  |  |  |  |  |  |  |  |  |
| 7 years | 4,090 | 30 (0.7%) | 6 (20.0%) | 10 (33.3%) | 4,158 | 14 (0.3%) | 6 (42.9%) | 4,130 | 82 (2.0%) | 10 (12.2%) |
| 10 years | 3,804 | 41 (1.1%) | 2 (4.9%) | 14 (34.1%) | 3,869 | 21 (0.5%) | 8 (38.1%) | 3,691 | 82 (2.2%) | 14 (17.1%) |
| 13 years | 3,429 | 31 (0.9%) | 6 (19.4%) | 10 (32.3%) | 3,497 | 14 (0.4%) | 6 (42.9%) | 3,185 | 48 (1.5%) | 10 (20.0%) |
| 15 years | 2,500 | 24 (1.0%) | 1 (4.2%) | 2 (8.3%) | 2,499 | 4 (0.2%) | 1 (25%) | 2,496 | 17 (0.7%) | 2 (11.8%) |
| **Females** |  |  |  |  |  |  |  |  |  |  |
| 7 years | 3,897 | 22 (0.6%) | 2 (9.0%) | 8 (36.4%) | 3,940 | 3 (0.08%) | 2 (66.6%) | 3,911 | 56 (1.4%) | 10 (17.9%) |
| 10 years | 3,756 | 33 (0.9%) | 2 (6.1%) | 7 (21.2%) | 3,805 | 12 (0.3%) | 4 (33.3%) | 3,662 | 78 (2.1%) | 7 (8.9%) |
| 13 years | 3,442 | 27 (0.8%) | 6 (22.2%) | 9 (33.3%) | 3,472 | 17 (0.5%) | 6 (35.3%) | 3,216 | 56 (1.5%) | 9 (19.6%) |
| 15 years | 2,785 | 62 (2.2%) | 12 (19.4%) | 20 (32.3%) | 2,782 | 34 (1.2%) | 12 (35.3%) | 2,779 | 84 (3.0%) | 20 (23.8%) |
