# Supplementary figures and images for: Correction: Validation of the Strengths and Difficulties Questionnaire (SDQ) emotional subscale in assessing depression and anxiety across development
Source: PLoS One. 2025 Jan 2;20(1):e0317151. doi: 10.1371/journal.pone.0317151 (PMC11694988; doi:10.1371/journal.pone.0317151)

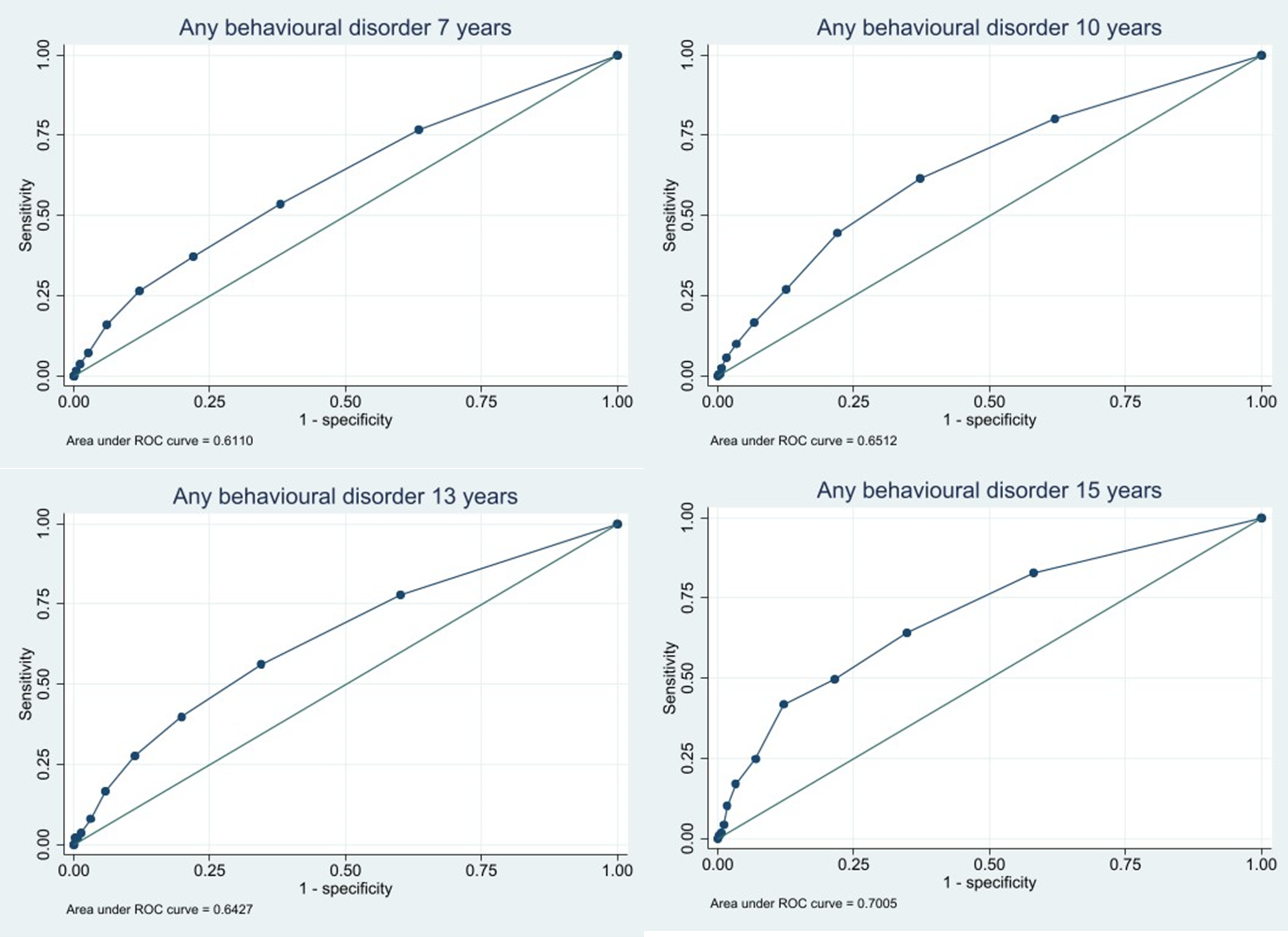

Supplement: S2 Fig — (TIF) [file pone.0317151.s001.tif]
